# Breast metastasis from EGFR/ALK negative lung adenocarcinoma: A case report

**DOI:** 10.1097/MD.0000000000023503

**Published:** 2020-12-04

**Authors:** Liyu Cao, Liting Lv

**Affiliations:** a Department of Medical Oncology; b Department of Oncology Surgery, Affiliated Dongyang Hospital of Wenzhou Medical University, Dongyang, Zhejiang, China.

**Keywords:** breast metastasis, lung adenocarcinoma

## Abstract

**Introduction:**

Lung adenocarcinoma is the most common type of lung cancer. Distant metastasis of lung adenocarcinoma often occurs in multiple organs. The common metastasis sites of lung cancer include the lungs, brain, bones, adrenal glands, and lymph nodes; however, breast metastasis is rare.

**Patient concerns:**

In this report, we describe a case of breast metastasis from lung adenocarcinoma. A 55-year-old woman reported left breast pain for more than 1 month.

**Diagnosis:**

Based on imaging, pathological examination, and immunohistochemical examination, the diagnosis of breast metastasis from lung adenocarcinoma was confirmed. Epidermal growth factor receptor mutations and anaplastic lymphoma kinase rearrangement were not detected by next-generation sequencing.

**Interventions:**

The patient was treated with six courses of a combination of albumin-bound paclitaxel, cisplatin, and bevacizumab over 21 days.

**Outcomes:**

After six cycles of palliative chemotherapy, her left breast pain and swelling subsided; in addition, her serum CA12-5, CYFRA, and CEA levels normalized by April 2019. PR status was evaluated as per the RECIST 1.1 criteria. The patient developed brain metastases 3 months later and died due to multiple organ failure.

**Conclusion:**

The possibility of breast metastasis should be considered in patients with existing malignant tumors and breast pain. Clinical and imaging examinations are helpful for diagnosis, and pathological and immunohistochemical analyses are the most important diagnostic tools.

## 1 Introduction

Lung adenocarcinoma is the most common type of lung cancer. Distant metastasis of lung adenocarcinoma to multiple organs occurs often and has poor prognosis. The common metastasis sites of lung cancer are the lungs, brain, bones, adrenal glands, and lymph nodes; however, breast metastasis is rare.^[1]^

It is estimated that 2.09 million new cases of lung cancer occurred globally in 2018, ranking first among all cancer types.^[2]^ Lung cancer is currently the leading cause of cancer deaths, accounting for nearly 20% of all cancer deaths.^[3,4]^

EGFR is a part of the ErbB family of transmembrane receptor tyrosine kinases, which mutate in some lung cancers.^[5]^ EML4-ALK fusion occurs in ∼3% to 5% of non-small cell lung cancer.^[6]^ In the presence of *EGFR* and/or *ALK* gene mutations, there is a choice between EGFR TKI drugs or ALK inhibitors for anti-tumor treatment. However, in the absence of the abovementioned gene mutations, traditional chemotherapy is the only treatment option.

Herein, we report a case of breast metastasis from lung adenocarcinoma and review the existing literature on the topic.

## 2 Case presentation

**Figure 1:**
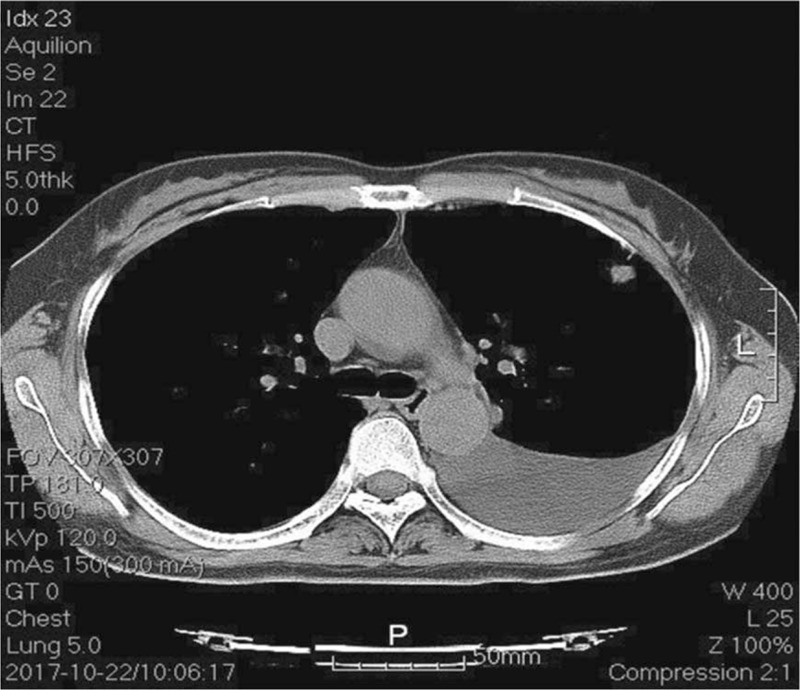
Computed tomography (CT): (1 axial CT images) results showed the presence of nodules in the left upper lobe and a small amount of pleural effusion on the left side in the lung.

A 55-year-old woman with no history of smoking was referred to our hospital in October 2017 with the chief complaint of cough that had lasted for a month. Physical examination did not indicate any abnormalities, and her condition was stable. Computed tomography (CT) (Fig. 1) of the chest revealed the presence of nodules in the left upper lobe of the lung and a small amount of pleural effusion on the left side.

Her serum tumor marker carbohydrate antigen 12-5 (CA12-5) level was 41.23 U/mL (normal: 35 U/mL), and her CYFRA level was 5.35 ng/mL (normal: 3.3 ng/mL); her CEA level was within the normal ranges (5.0 ng/mL).

**Figure 2:**
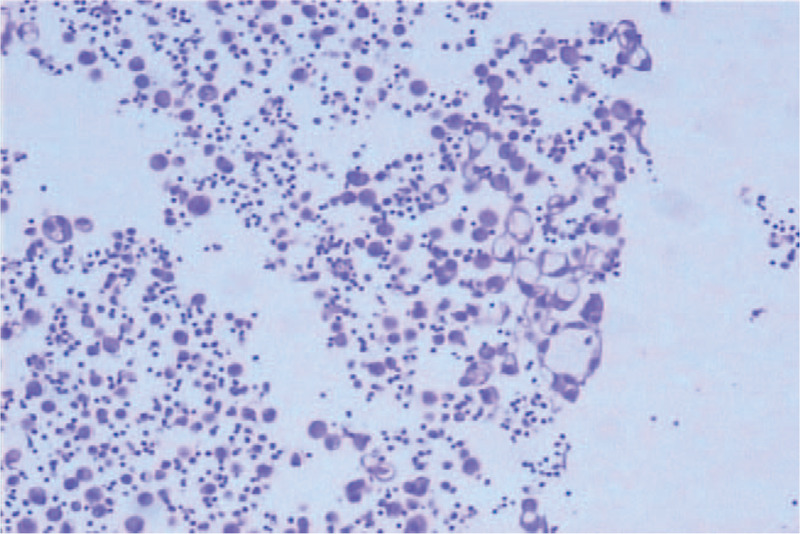
The immunohistochemical diagnosis of pleural effusion (original magnification ×400). The pleural effusion cell wax mass was showed as adenocarcinoma and considering lung origin in combination with clinical and immunohistochemical findings.

Thoracic puncture and catheter drainage were performed on October 25, 2017. Combined clinical and immunohistochemical analysis led to the pathological (Fig. 2) diagnosis of (pleural fluid cell) adenocarcinoma, with the lung considered as point of origin. Immunohistochemical examination revealed positivity for naspin-A, CEA, calretinin mesothelial cells, cytokeratin (CK)7, transcription termination factor 1 (TTF-1), and Hector Battifora mesothelial-1 (HBME-1) and negativity for CK5/6 and P40. Furthermore, PET-CT confirmed the diagnosis of lung adenocarcinoma with malignant pleural effusion (cT × N × M1, stage IV). Molecular testing (circulating tumor ctDNA by high-throughput next-generation sequencing) revealed no EGFR mutations or ALK rearrangements.

She was treated with 30 mg/m^2^ lobaplatin administered intrathoracically. She then received 6 cycles of systemic chemotherapy combined with targeted therapy, including 500 mg/m^2^ pemetrexed (day 1), carboplatin (AUC 5, day 1), and 7.5 mg/kg bevacizumab (day 1) over 21 days, followed by 10 courses of maintenance therapy with 500 mg/m^2^ pemetrexed (day 1) and 7.5 mg/kg bevacizumab (day 1) for 21 days.

At the 1-year follow-up, the patient reported experiencing left breast pain for more than 1 month; however, the breast appeared abnormal. Physical examination revealed tenderness of the entire left breast, with thickened skin. Her serum CA12-5 level was 61.2 U/mL, CYFRA level was 11.3 ng/mL, and CEA level was 5.08 ng/mL.

**Figure 3:**
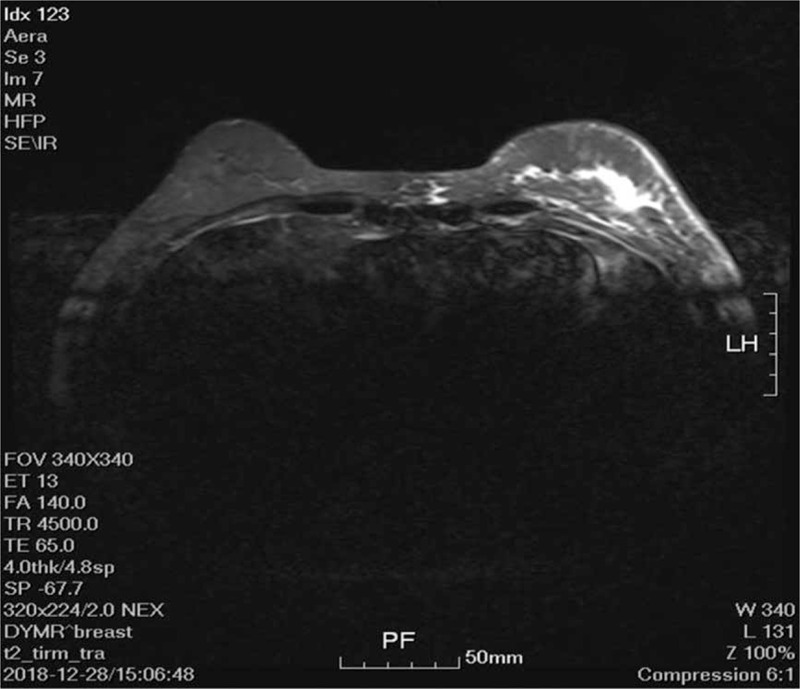
Nuclear magnetic resonance imaging (MRI): (1 axial MR images) results showed a left-breast mass patchy enhancement lesion and left axillary lymph node enlargement.

Furthermore, nuclear magnetic resonance imaging (Fig. 3) of the breasts showed a left-breast mass patchy enhancement lesion with a breast imaging, reporting and data system level 5 (BI-RADS 5) and left axillary lymph node enlargement. Color doppler ultrasound breast + color ultrasound axillary lymph nodes revealed flake hypoechoic glands in the upper quadrant of the left nipple and the outer upper quadrant, with the dimensions 51 × 12 × 43 mm, belonging to six types of BI-RADS. There were several hypoechoic nodules in the left armpit; the largest was ∼10 × 14 mm in size, and the possibility of a metastatic tumor was considered.

**Figure 4:**
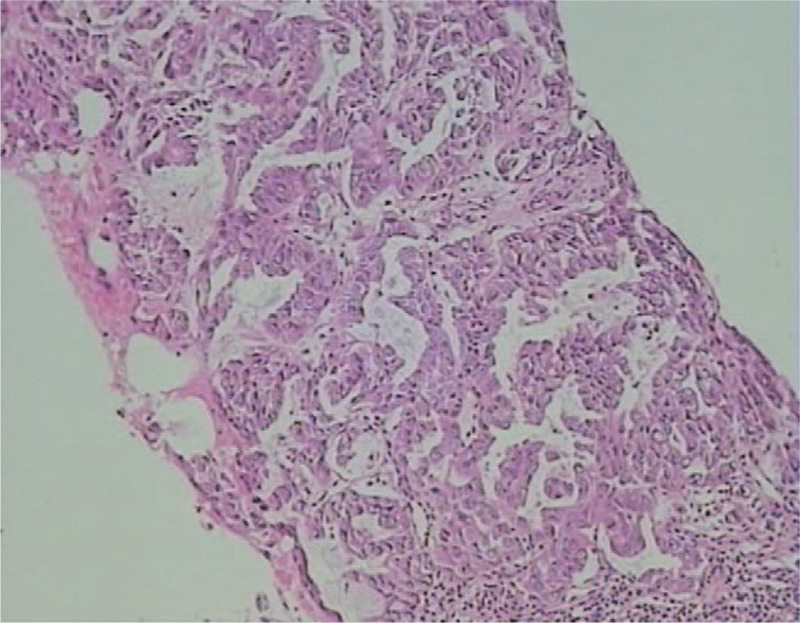
The immunohistochemical diagnosis of the left breast (original magnification ×400). Left breast puncture showed invasive carcinoma and metastasis of lung adenocarcinoma according to immunohistochemistry.

A hollow needle biopsy of the left breast and axillary lymph node was performed, which revealed invasive ductal carcinoma showing positivity for CK7, TTF-1, napsin-A (weak), E-Ca3, and 20% Ki-67 and negativity for estrogen receptor (ER), progesterone receptor (PR), c-erbB-2, P63, and GATA-3 (Fig. 4), confirming breast metastasis of lung cancer.

The patient was treated with six courses of a combination of 260 mg/m^2^ albumin-bound paclitaxel (day 1), 75 mg/m^2^ cisplatin (day 1), and 7.5 mg/kg bevacizumab (day 1) over 21 days. The results of the two cycles were reviewed and showed that the lamellar hypoechoic lesion was in the gland of in upper outer quadrant of the left papilla, within a range of 27 × 24 × 10 mm. The BI-RADS classification was 6. There were several hypoechoic nodules in the left armpit, and the largest one was ∼14 × 10 mm. partial response status was evaluated as per the RECIST 1.1 criteria. After 4.5 months of treatment, her left breast pain and swelling subsided, and her serum CA12-5, CYFRA, and CEA levels normalized by April 2019. The effect of B ultrasound was continuous PR. During this period, we regularly performed chest CT to ensure that the pulmonary lesions was stable.

Three months later, the patient developed dizziness and headache. Nuclear magnetic resonance imaging revealed multiple nodules on both sides of the brain, confirming brain metastases. She received 1 cycle of temozolamide (150 mg/m^2^, 28-day cycle). Then, a month later, she died. The cause of death was multiple organ failure.

### 2.1 Ethical approval and consent

This case report was approved by the Ethics Committee of Dongyang People's Hospital. Written informed consent was obtained from the patient for publication of this clinical case report.

## 3 Discussion

Primary pulmonary adenocarcinoma is a common malignant tumor that has emerged as one of the tumors with the highest mortality rates. Metastasis of lung cancer is one of the most difficult problems impacting clinical treatment and affects the prognosis of patients.^[7–9]^ Breast metastasis secondary to extramammary malignant tumors is very rare, with an incidence of ∼0.4% to 1.3% in breast cancer cases.^[10]^ Lee et al^[11]^ reported 33 cases of breast metastasis from extramammary malignant tumors, of which the most common primary tumor was gastric cancer. However, Luo et al^[12]^ reported 24 cases of breast metastasis from extramammary malignant tumors, the most common primary malignancy in the extramammary lesions was lung cancer.

Studies have shown that breast metastasis is characterized by painless breast nodules that grow rapidly. Skin changes and nipple discharge are rare. Of the patients, 26% had either bilateral or unilateral multiple breast nodules, and only 4% had diffuse breast changes.^[13–15]^ However, there have been no reports of breast pain being the first symptom.

Metastases to the breast from extramammary neoplasms can occur via both hematologic and lymphatic routes.^[16,17]^ Ipsilateral breast metastasis from lung adenocarcinoma can also occur via the lymphatic route. Huang et al^[18]^ proposed that lung cancer cells seed on the pleura, invade axillary lymph nodes, and metastasize to the ipsilateral breast through retrograde lymphatic vessels. In such cases, patients present with ipsilateral pleural effusion thickening, axillary lymph node enlargement, and ipsilateral breast metastasis. Hong et al^[19]^ reported a series of lung cancer cases with contralateral breast metastasis. The reason for this may be that the tumor cells enter the venous and systemic circulation along the lymphatic circulation through the thoracic duct to reach the breast or tumor cells; they enter the blood directly causing distant metastasis.

Differentiating between primary breast cancer and breast metastases is challenging. For adenocarcinomas with a similar histomorphology to that of primary breast tumors, a differential diagnosis should be made by immunohistochemical examination.^[20,21]^ CK7 analysis is useful in determining the origin of metastatic lesions and strongly indicated that all samples from our patient were of either breast or lung origin.^[22]^ Although thyroid TTF-1 is expressed in 68% to 76% of lung adenocarcinomas, positivity in breast adenocarcinoma has never been reported.^[23]^

Napsin A is expressed in 84% of primary lung adenocarcinomas but not in other adenocarcinoma types.^[24]^ In our case, the biopsy of breast tissue was positive for CK7, TTF-1, and napsin A, suggesting that the cancer had originated in the lung. The expression level of ER in patients with lung cancer is very low.^[25]^ In our case, immunohistochemical analysis of the breast tissue biopsy was performed and the expression of ER and PR confirmed that the cancer was not primary breast cancer.

EGFR belongs to the ErbB family of receptor tyrosine Kinase.^[26]^ Increased EGFR expression in the primary tumor is associated with unregulated proliferation, malignant transformation, metastasis, and apoptosis resistance of cancer cells.^[27,28]^ EGFR is a crucial triple-negative breast cancer (TNBC) biomarker that is upregulated in ∼60% of TNBC cases.^[29]^ Some studies have found no activating EGFR mutations in TNBC patients,^[30–32]^ whereas others have reported that 3% to 11% of TNBC harbor EGFR mutations.^[33,34]^ The variability in the results might be because of the processing methods used or geographic or ethnic differences. In patients with lung cancer, *ALK* gene mutations (EML4-ALK) caused by chromosomal inversion promoted the occurrence and progression of lung cancer.^[35,36]^ EGFR TKI drugs and ALK inhibitors can be used to treat patients with advanced lung cancer with *EGFR* gene mutations and ALK fusion mutations. For EGFR/ALK negative patients, anti-tumor treatments still rely on the traditional chemotherapy.

In the future, more attention should be paid to EGFR mutations and ALK rearrangements in primary and metastatic tumors to allow for accurate diagnosis and personalized, precise medication in clinical practice. In our case, no EGFR mutation or ALK rearrangement was found in the primary lung lesions or breast metastases.

In conclusion, the possibility of breast metastasis should be considered in patients with existing malignant tumors and breast pain. Clinical and imaging examinations are helpful for diagnosis, and pathological and immunohistochemical analyses are the most important diagnostic tools.

## Author contributions

**Data curation:** Liting Lv.

**Formal analysis:** Liyu Cao, Liting Lv.

**Funding acquisition:** Liyu Cao.

**Writing – original draft:** Liyu Cao, Liting Lv.

**Writing – review & editing:** Liyu Cao.
